# Adherence to Growth Hormone Treatment Using a Connected Device in Latin America: Real-World Exploratory Descriptive Analysis Study

**DOI:** 10.2196/32626

**Published:** 2022-01-20

**Authors:** Aria Assefi, Paula van Dommelen, Lilian Arnaud, Carlos Otero, Luis Fernandez-Luque, Ekaterina Koledova, Luis Eduardo Calliari

**Affiliations:** 1 Fertility and Endocrinology Merck SA (an affiliate of Merck KGaA, Darmstadt, Germany) Buenos Aires Argentina; 2 Department of Child Health The Netherlands Organization for Applied Scientific Research TNO Leiden Netherlands; 3 Global Healthcare Operations, Connected Health & Devices Ares Trading SA (an affiliate of Merck KGaA, Darmstadt, Germany) Eysins Switzerland; 4 Departamento de Informática en Salud Hospital Italiano de Buenos Aires Buenos Aires Argentina; 5 Adhera Health Inc Palo Alto, CA United States; 6 Global Medical Affairs Cardiometabolic & Endocrinology the healthcare business of Merck KGaA Darmstadt Germany; 7 Pediatric Endocrinology Unit Pediatric Department Santa Casa de São Paulo School of Medical Sciences São Paulo Brazil

**Keywords:** adherence, connected device, growth, growth disorders, growth hormone, electronic injection device, real-world data, disorder, therapy, treatment, children, outcome

## Abstract

**Background:**

Recombinant human growth hormone (rhGH) therapy is an effective treatment for children with growth disorders. However, poor outcomes are often associated with suboptimal adherence to treatment.

**Objective:**

The easypod connected injection device records and transmits injection settings and dose data from patients receiving rhGH. In this study, we evaluated adherence to rhGH treatment, and associated growth outcomes, in Latin American patients.

**Methods:**

Adherence and growth data from patients aged 2-18 years from 12 Latin American countries were analyzed. Adherence data were available for 6207 patients with 2,449,879 injections, and growth data were available for 497 patients with 2232 measurements. Adherence was categorized, based on milligrams of rhGH injected versus milligrams of rhGH prescribed, as high (≥85%), intermediate (>56%-<85%), or low (≤56%). Transmission frequency was categorized as high (≥1 per 3 months) or low (<1 per 3 months). Chi-square tests were applied to study the effect of pubertal status at treatment start and sex on high adherence, and to test differences in frequency transmission between the three adherence levels. Multilevel linear regression techniques were applied to study the effect of adherence on observed change in height standard deviation score (∆HSDS).

**Results:**

Overall, 68% (4213/6207), 25% (n=1574), and 7% (n=420) of patients had high, intermediate, and low adherence, respectively. Pubertal status at treatment start and sex did not have a significant effect on high adherence. Significant differences were found in the proportion of patients with high transmission frequency between high (2018/3404, 59%), intermediate (608/1331, 46%), and low (123/351, 35%) adherence groups (*P*<.001). Adherence level had a significant effect on ∆HSDS (*P*=.006). Mean catch-up growth between 0-24 months was +0.65 SD overall (+0.52 SD in patients with low/intermediate monthly adherence and +0.69 SD in patients with high monthly adherence). This difference translated into 1.1 cm greater catch-up growth with high adherence.

**Conclusions:**

The data extracted from the easypod Connect ecosystem showed high adherence to rhGH treatment in Latin American patients, with positive growth outcomes, indicating the importance of connected device solutions for rhGH treatment in patients with growth disorders.

## Introduction

Adherence to long-term pharmacological treatments, such as growth hormone (GH) therapy for growth disorders, is an area with great potential for improvement [1,2]. Poor long-term adherence to GH treatment is known to affect final adult height and additional clinical outcomes in children with growth disorders [3]. Moreover, enthusiasm and motivation to adhere to treatment may decrease over time because the long-term benefits of GH treatment are not immediately obvious to children, and administering daily subcutaneous injections places a significant burden on them and their parents/caregivers [3-5]. Indeed, adherence to GH treatment has been shown to be statistically significantly higher in treatment-naive children compared with those experienced in their treatment [4].

Adherence to GH treatment in the real-world setting has always been difficult to monitor, given the use of unreliable proxy methods such as patient testimony or records of prescriptions filled/vials counted [4,5]. Furthermore, detection of poor adherence to GH treatment can be problematic because patients/caregivers may be reluctant to admit to (or do not remember) missed doses and may overestimate their adherence to treatment during discussions with health care providers (HCPs) [4]. Devices that offer a dose-setting memory may therefore be beneficial in improving adherence [6]. In addition, with prevalence estimates of nonadherence ranging from 5% to 82% [5], it is difficult to compare adherence rates among studies due to the variability in methods used to evaluate and define adherence [5,7]. Lastly, studies assessing adherence are sometimes constrained by low patient numbers and there is often only one participating center, which limits the extrapolation of results to different settings [8].

Automatic transmission of injection data provides a more accurate insight into real-world adherence patterns and enables HCPs to potentially eliminate poor adherence as a reason for a suboptimal response to GH treatment [4,9,10]. The use of a connected injection device to deliver GH treatment limits the risk of misreporting or faulty recall of adherence, and allows HCPs to accurately monitor their patients’ real-world adherence behavior over time [9]. Patient confidentiality is maintained because only the treating HCPs and patient support programs (PSPs) can access patients’ complete data from a secure cloud-based database, and only deidentified (pseudonymized) data are used to generate aggregated and anonymized results for research purposes. A connected injection device thereby enables HCPs to access the transmitted data and gain insights into both individual and overall patterns of adherence to GH treatment [9,11].

As different health care systems and variations in clinical practice around the world may affect adherence locally, the deployment of a connected injection device for the treatment of growth disorders across different countries allows the study of behavioral adherence patterns across populations and longitudinally for thousands of patients. This substantial compendium of patient-generated data has also been applied in the context of understanding patterns in diabetes thanks to glucose monitoring technologies [12,13]. In terms of monitoring adherence in large cohorts, oral medication use has been monitored using “smart pillboxes” [14] and “smart pill bottles” [15,16].

Global analysis of real-world data obtained from connected injection devices for GH treatment has shown that children with high adherence were most likely to regularly transmit data, and that prepubertal children showed higher adherence than older children and adolescents [17]. This analysis showed the potential of developing a global adherence decision support system (ADSS) by analyzing trends in real-world adherence data, but did not include insights from different health care systems.

The Latin American region is one of the fastest growing regions in terms of the adoption of digital health [18,19]. This has facilitated the implementation of a web-based platform connected with injection devices for GH treatment across several countries in Latin America, a region in which digital health studies have previously been lacking. The objective of this study, therefore, was to evaluate real-world adherence to recombinant human GH (rhGH) therapy administered via a connected injection device in one specific region (Latin America) to provide an update to an earlier, smaller Latin American analysis, previously published only in abstract form [11]. Additionally, we studied catch-up growth and its association with adherence in a subgroup of patients.

## Methods

### Patient Population

In this analysis, we included children with growth disorders from 12 Latin American countries (Argentina, Brazil, Chile, Colombia, Costa Rica, Dominican Republic, El Salvador, Guatemala, Mexico, Nicaragua, Panama, and Peru) and assessed the effects of pubertal status at treatment start, sex, and engagement with treatment on their adherence. An analysis of longitudinal records for a total of 13,553 children in the global database was recently published [17], but here we focus only on data from patients in these Latin American countries. The total data set available in the global database comprises data from the 5-year global Easypod Connect Observational Study (ECOS) [9] and from all patients worldwide who have received treatment with rhGH (somatropin [Saizen]; the healthcare business of Merck Healthcare KGaA, Darmstadt, Germany) administered via the easypod connected injection device.

### A Digital Ecosystem for Supporting and Monitoring Adherence to rhGH Therapy

The easypod electromechanical injection device, in combination with the easypod Connect web-based platform, as part of an ADSS [20], electronically records accurate, objective details of the date, time, and dose of injections for patients receiving rhGH for the treatment of growth disorders [21]. All of these data are recorded by the device and stored internally for up to 3 years. The patient can then transmit these data to the easypod Connect platform and a secure internet cloud-based database [9].

### Study Design and Inclusion Criteria

**Figure 1:**
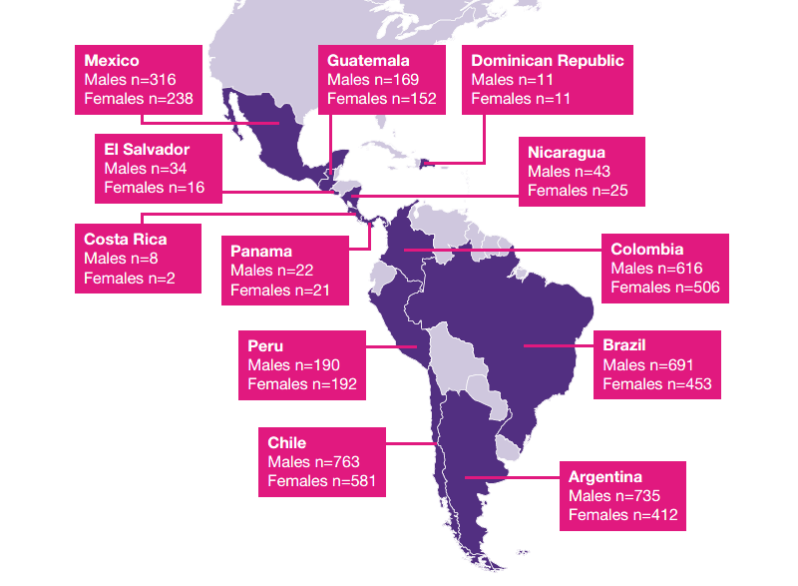
Participating Latin American countries.

This was an exploratory descriptive analysis study during which 4 years of adherence data were analyzed from 6207 pediatric patients with 2,449,879 prescribed injections of rhGH delivered via easypod to treat growth disorders, and who were transmitting data to the easypod Connect system between January 2007 and December 2020. These patients resided in 12 Latin American countries (Figure 1). To avoid inclusion of test doses or training injections, only data after the 10th injection registered for each individual were analyzed. Data were downloaded from the easypod Connect platform in January 2021, but the period of recorded data varied according to each individual patient’s treatment duration. Transmission data from January 2016 to December 2020 were used. We selected patients who were aged 2-18 years at treatment start.

Eligible patients from each of the participating countries had been enrolled in the database and attended at least one visit, according to local routine clinical practice. Diagnoses and decisions on treatment were made at the discretion of the physician responsible for each patient, following standard endocrinologic practice for each of the participating countries. Prior to enrollment, patients/caregivers reviewed and voluntarily signed an informed consent form materializing their agreement for data collection, storage, and use of their child’s pseudonymized data to create aggregated statistical and general adherence reports.

For height, we selected patients with at least two measurements. Adherence (calculated as milligrams of rhGH injected versus milligrams of rhGH prescribed) was categorized as high (≥85%), intermediate (>56%-<85%), or low (≤56%) for patients on either 6 or 7 injections per week, the two possible regimens for treatment with rhGH. The dosage and frequency of rhGH therapy as per easypod settings were defined by HCPs and data transmissions were initiated by the child, parent/caregiver, or HCP. Adherence was assessed overall and monthly, and explored by puberty status at treatment start (nominal cutoffs at 10 years for girls, and 12 years for boys), sex, and transmission frequency (defined as the total number of transmissions divided by the duration of treatment, and categorized as high [≥1 per 3 months] versus low [<1 per 3 months]) in the selected patients with available adherence data for ≥3 months between January 2016 and December 2020. Transmission frequency was calculated in each adherence category as a proxy measure of the patient’s engagement in disease management using easypod. No imputation was made for missing data or withdrawal from the study.

### Patient Data and Calculations

Height data were available for 497 patients with 2232 measurements; this included 355 patients from Argentina, 64 from Brazil, 70 from Guatemala, and 8 from Mexico. Height standard deviation scores (HSDS) were calculated using the World Health Organization references [22,23]. Linear interpolation between height measurements was applied to calculate monthly catch-up growth (∆HSDS) overall, and by low/intermediate versus high adherence, and within the subgroup of patients (n=40) aged <8 years with HSDS of <–2, for which we assume an optimal catch-up growth. The cutoff of 8 years was chosen so the definition of short stature, age, and treatment duration ensured this would be before the start of puberty in both genders. Cubic smoothing splines were fitted to obtain the curves for catch-up growth between 0-24 months.

### Statistical Analysis

Descriptive statistics were used to describe differences over time in adherence (low, intermediate, or high), puberty status (prepubertal or pubertal), and sex. Chi-square tests were applied to test differences in high adherence between girls and boys, and between prepubertal and pubertal girls and boys. In addition, a Chi-square test was applied to test differences in high frequency transmission between the high, intermediate, and low adherence groups. Multilevel linear regression techniques were applied to study the effect of adherence level on ∆HSDS between all observed growth measurements, adjusted for the time intervals between them.

### Ethical Considerations

Treatment with rhGH via easypod was conducted according to local practice. This real-world, retrospective analysis of the data set was performed in accordance with the informed consent form, signed by caregivers of children and adult patients materializing their agreement for data collection, storage, and use of their pseudonymized data to create aggregated statistical and general adherence reports.

### Data Availability Statement

Any requests for data by qualified scientific and medical researchers for legitimate research purposes will be subject to the healthcare business of Merck KGaA’s Data Sharing Policy. All requests should be submitted in writing to the healthcare business of Merck KGaA’s data sharing portal [24]. When the healthcare business of Merck KGaA has a coresearch, codevelopment, or comarketing or copromotion agreement, or when the product has been outlicensed, the responsibility for disclosure might be dependent on the agreement between parties. Under these circumstances, the healthcare business of Merck KGaA will endeavor to gain agreement to share data in response to requests.

## Results

### Patient Population and Demographics

**Figure 2:**
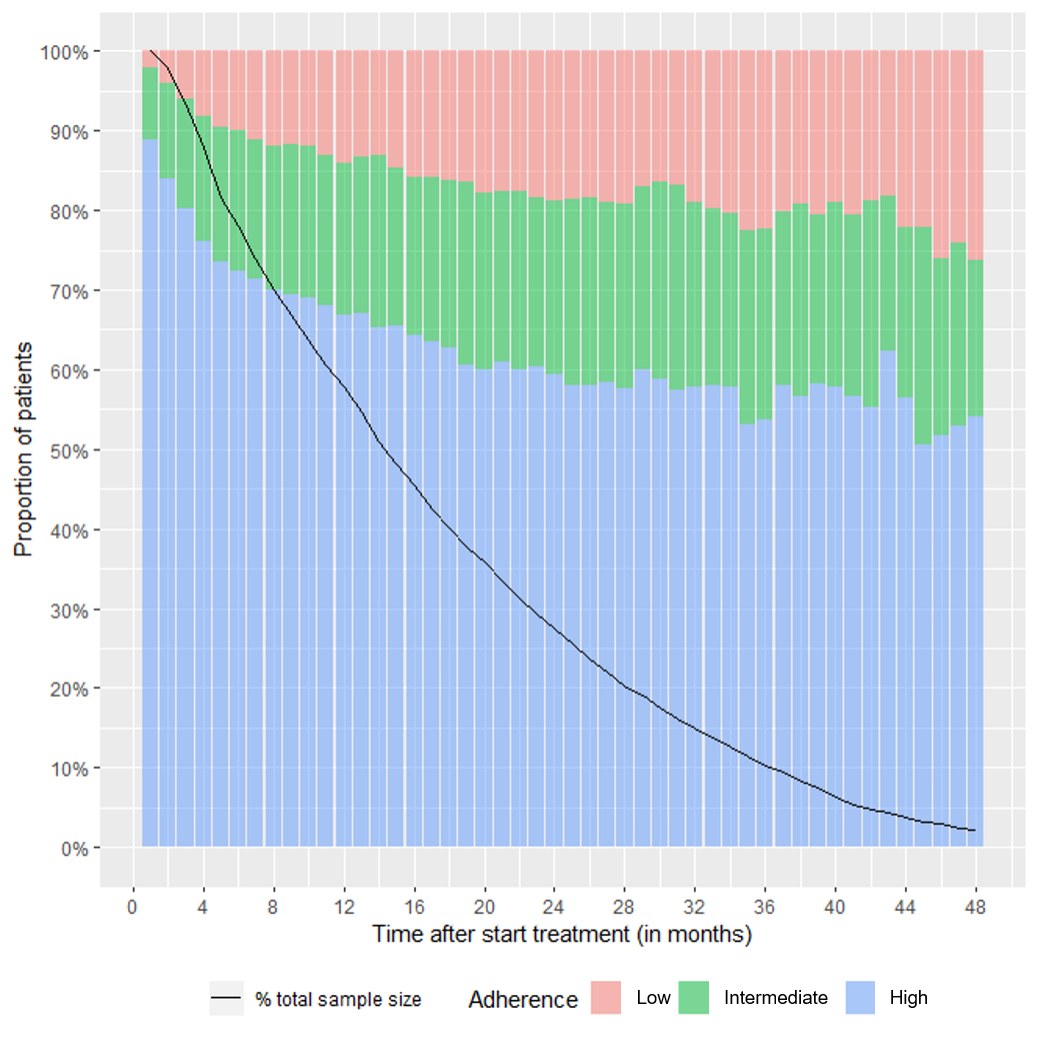
Proportion of patients who were adherent at each time point. Adherence was recorded for the cross-section of children or their caregivers transmitting data at each time point; no imputation was made for missing data or withdrawal from the study.

Complete data were available for 6207 patients, where “overall” is defined here (and throughout) as the total number of patients who received rhGH, started treatment at age 2-18 years, and for whom data were available. Transmission data were available for 5086 patients. Patient demographics according to adherence rates are presented for this data set in Table 1. The number of patients decreased from 6207 patients in the first month (100%), to 3594 patients (58%) at month 12, to 1707 patients at month 24, and to <600 patients (<10%) after month 36 (Figure 2; black line). Growth data were available for 497 patients overall, and decreased to 330 patients at months 13-24, 150 patients at months 25-36, and 37 patients at months 37-48.

**Table 1:** Overall patient demographics according to adherence rates.

Characteristic	Adherence^a^				Total (N=6207)
	High (n=4213)	Intermediate (n=1574)	Low (n=420)		
Mean age (SD) of boys at start, years	10.6 (3.4)	10.9 (3.3)	10.6 (3.4)	10.7 (3.3)	
Boys aged <12 years at start, n (%)	1424 (68)	526 (25)	157 (7)	2107 (34)	
Boys aged ≥12 years at start, n (%)	985 (66)	394 (26)	112 (8)	1491 (24)	
Mean age (SD) of girls at start, years	9.7 (2.8)	10.0 (2.8)	9.8 (3.2)	9.8 (2.8)	
Girls aged <10 years at start, n (%)	839 (69)	308 (25)	72 (6)	1219 (20)	
Girls aged ≥10 years at start, n (%)	965 (69)	346 (25)	79 (6)	1390 (22)	

^a^Adherence was categorized as high (≥85%), intermediate (>56%-<85%), or low (≤56%).

### Adherence Overall and Over Time

Overall, 68% (4213/6207) of patients were in the high adherence category, 25% (n=1574) were in the intermediate adherence category, and 7% (n=420) were in the low adherence category. Furthermore, at each time point, there was a higher proportion of patients in the high adherence category than in the intermediate and low categories combined (Figure 2). High adherence decreased from 89% (5514/6207) to 59% (1013/1707) between 1-24 months, and ranged from 50%-62% between 25-48 months. However, despite there being a decrease in the proportion of patients in the high adherence category over time, 67% (2399/3594) and 59% (1013/1707) of patients were still in the high adherence category at months 12 and 24, respectively.

### Effect of Age, Sex, and Engagement With the Easypod Device on Adherence

**Figure 3:**
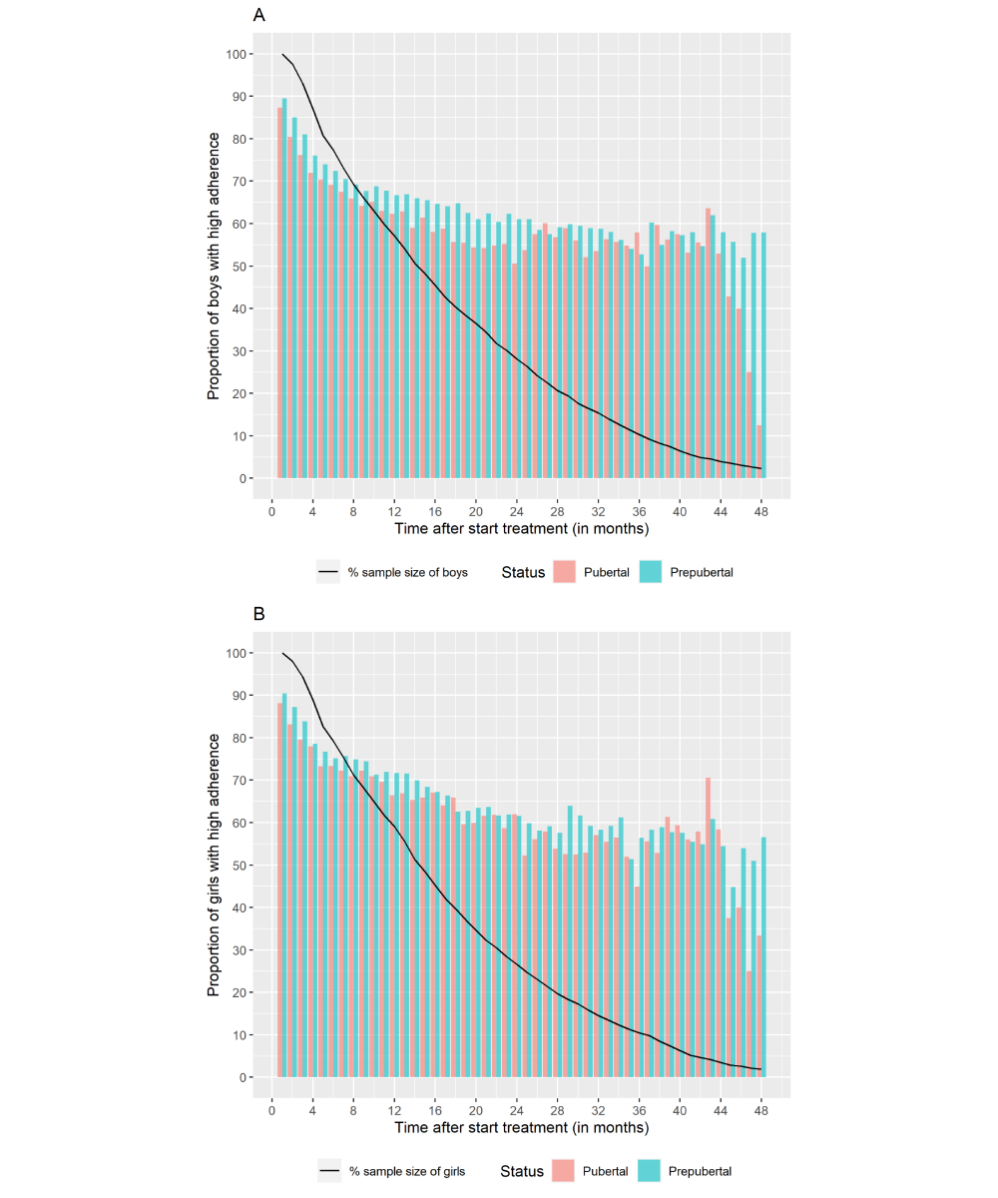
Adherence at each time point stratified by nominal pubertal age and sex (A: boys, B: girls). Adherence was recorded for the cross-section of children or their caregivers transmitting data at each time point; no imputation was made for missing data or withdrawal from the study.

There were no significant differences in high adherence between boys (2409/3598, 67%) and girls (1804/2609, 69%) or between prepubertal and pubertal patients (1424/2107, 68% versus 985/1491, 66% in boys, both 69% [839/1219; 965/1390] in girls, respectively). Figures 3A and 3B show adherence at each time point stratified by nominal pubertal age and sex. Figure 3A shows a larger proportion of prepubertal boys with high adherence between 1-25 months compared with pubertal boys. For the majority of these months (17/25), the proportion of high adherence was significantly (*P*<.05) higher in prepubertal boys compared with pubertal boys.

In addition, there were significant differences in the proportion of patients with high transmission frequency between the adherence groups; 59% (2018/3404), 46% (608/1331), and 35% (123/351) in the high, intermediate, and low adherence groups, respectively (*P*<.001).

### Between-Country Variation

The proportion of children in the high adherence category varied by country, ranging from 45% (10/22) in the Dominican Republic to 82% (943/1144) in Brazil (Table 2).

**Table 2:** Adherence rates by age, sex, and country.

Country and adherence^a^		Boys aged <12 years, n	Boys aged ≥12 years, n	Girls aged <10 years, n	Girls aged ≥10 years, n	Total number of patients with ≥1 injection		Adherence ≥85%, n (%)	
**Argentina**							1147		759 (66)
	High	322	163	151	123				
	Intermediate	113	72	66	41				
	Low	46	19	18	13				
**Brazil**							1144		943 (82)
	High	347	225	169	202				
	Intermediate	68	39	35	35				
	Low	9	3	6	6				
**Chile**							1344		949 (71)
	High	291	242	176	240				
	Intermediate	96	84	44	95				
	Low	17	33	12	14				
**Colombia**							1122		625 (56)
	High	170	152	149	154				
	Intermediate	125	108	81	86				
	Low	39	22	18	18				
**Costa Rica**							10		8 (80)
	High	4	3	0	1				
	Intermediate	0	1	1	0				
	Low	0	0	0	0				
**Dominican Republic**							22		10 (45)
	High	1	3	3	3				
	Intermediate	2	0	0	4				
	Low	2	3	0	1				
**El Salvador**							50		27 (54)
	High	7	12	5	3				
	Intermediate	8	5	2	3				
	Low	1	1	2	1				
**Guatemala**							321		221 (69)
	High	71	38	43	69				
	Intermediate	26	14	18	16				
	Low	8	12	0	6				
**Mexico**							554		355 (64)
	High	113	83	72	87				
	Intermediate	48	40	32	33				
	Low	23	9	6	8				
**Nicaragua**							68		33 (49)
	High	13	8	5	7				
	Intermediate	8	7	5	4				
	Low	6	1	2	2				
**Panama**							43		23 (53)
	High	6	5	4	8				
	Intermediate	5	3	3	6				
	Low	2	1	0	0				
**Peru**							382		260 (68)
	High	79	51	62	68				
	Intermediate	27	21	21	23				
	Low	4	8	8	10				

^a^Adherence was categorized as high (≥85%), intermediate (>56%-<85%), or low (≤56%).

### Catch-up Growth

**Figure 4:**
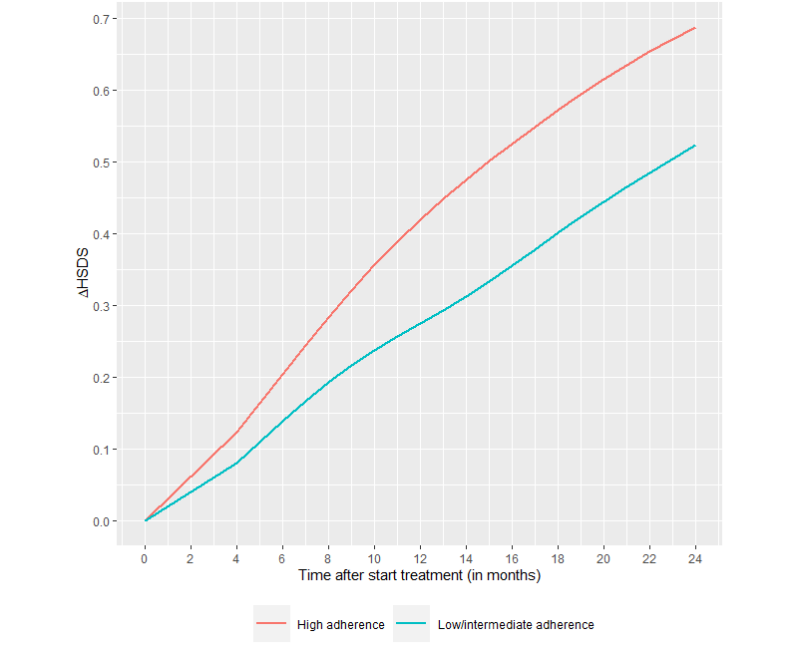
Catch-up growth between 0-24 months stratified by low/intermediate adherence (<85%) and high adherence (≥85%). ∆HSDS: change in height standard deviation score.

Figure 4 shows the mean catch-up growth (∆HSDS) between 0-24 months, stratified by high versus intermediate/low adherence. Adherence level (low/intermediate versus high) had a significant effect on ∆HSDS (*P*=.006). Mean catch-up growth between 0-12 months was +0.39 SD overall, with +0.27 SD in patients with low/intermediate monthly adherence and +0.42 SD in patients with high monthly adherence. Mean catch-up growth between 0-24 months was +0.65 SD overall, with +0.52 SD in patients with low/intermediate monthly adherence and +0.69 SD in patients with high monthly adherence. Mean catch-up growth within the subgroup of patients (n=40) aged <8 years and HSDS <–2 at start was +1.03 SD. It was not possible to stratify this subgroup of patients by low and high adherence due to the small sample size.

## Discussion

### Principal Results

The data extracted from the easypod Connect ecosystem showed that children receiving rhGH therapy demonstrated high adherence over 4 years in a real-world setting in 12 Latin American countries.

Use of easypod Connect, which showed high adherence in patients with high transmission rates, could be regarded as a proxy of usability and utility of the system by patients and HCPs. In such large-scale deployments of digital health systems, usability cannot be easily measured using questionnaires as this would pose a major user/participant burden. Instead, transmissions and data usage can be used toward identifying patterns of usage of the system and adherence to treatment. Indeed, in this regard, a framework has been proposed to guide future implementation and research on the use of digital health tools to support patients with growth disorders who require GH therapy [25]. These insights can be further investigated in smaller-scale studies (eg, exploring qualitative issues with interviews).

We observed that patients who transmitted their data tended to have higher adherence; therefore, a potential hypothesis to further explore is the impact of patients knowing that their adherence data is being observed and is useful to their HCP. Studies in other therapeutic areas have found that factors related to the Theory of Planned Behavior can predict ~50% variance in adherence [26]. Future studies can look into the behavioral impact of monitoring adherence and the role this plays in the interaction of HCPs with the data during clinical visits (eg, clinicians reviewing adherence data during the patient visit, how the system is introduced to the patient).

### Comparison With Other Studies

The selected data from this regional study are broadly consistent with recently published data from the global easypod Connect database, which demonstrated that, of 13,553 children, 71% were in the high adherence category [17], and with a previous exploratory analysis (68% in the high adherence category) [27]. Children with high adherence were most likely to regularly transmit data, reflecting engagement with the easypod device [17]. Furthermore, the ECOS has shown that the majority (62%) of patients maintained an observed adherence rate of ≥80% to rhGH therapy over 3 years of easypod use [9]. Similarly, a preliminary analysis evaluated real-world adherence to rhGH therapy administered via easypod over 1, 3, 6, and 12 months in Latin American patients [11]. Analysis of data extracted in February 2018 from 2727 patients transmitting their injection data to easypod Connect showed that the majority (67%) were still in the high adherence category (defined as ≥85%) at 12 months, with girls (64% versus 63% of boys) and prepubertal patients (69% versus 60% of pubertal patients) being the most adherent [11].

In both the preliminary Latin American study and other analyses, the proportion of children with high adherence declined over time, and factors such as sex and nominal age at puberty appeared to have only a small effect on adherence rates [11,17]. Similarly, in this study, after 12 months, 33% (n=1195 out of N=3594) of Latin American patients were in the low or intermediate adherence categories, while this was 11% (n=693 out of N=6207) in the first month of treatment. Additionally, a slightly higher proportion of girls overall were in the high adherence category compared with boys, as were patients who were prepubertal at treatment start versus pubertal patients; however, overall, these results were not statistically significant. These results demonstrate that adherence is an issue that needs to be addressed continuously by HCPs and included in discussions with patients and their families/caregivers, taking both patient sex and pubertal status into account.

Patient attrition over time may be due to a number of factors. Patients may have been switched to a different rhGH, may have continued taking somatropin rhGH using a pen injector, may have continued taking rhGH using easypod but without performing any further data transmission, or may have stopped treatment completely. Minimizing patient attrition is an important consideration in long-term clinical trials, and determining the predictors of attrition is key to identifying patients at risk of missed visits or dropout; such patients may be excluded from a trial or efforts may need to be made to prevent their subsequent dropout once enrolled [28]. This is especially important in the case of growth disorders since rhGH treatment is required over the long term and requires good adherence to achieve optimal outcomes. Indeed, determining predictors of attrition and using early trial retention strategies (eg, management of reluctant or hard-to-locate study participants) have led to improved attrition rates in studies of asthma and behavioral disorders [28,29]. Thus, such approaches could be considered in future studies investigating adherence to rhGH treatment.

Indeed, previously reported individual cases of patients receiving rhGH have indicated that direct access to adherence monitoring by HCPs followed by intervention can make a difference to a patient’s management and motivation [30-32]. The authors’ personal experience shows that, in many cases, families are not always aware that their child’s adherence is suboptimal and are surprised by this information when informed by the HCP of the data recorded by easypod. This information is key because it can explain a smaller growth catch-up without the need for further investigation into other potential causes. In this regard, devices with a dose setting were the preferred choice among patients and caregivers in a recent study by Tanaka et al [6].

Optimizing adherence might also be achieved through the use of structured and active interventions from HCPs, patient/caregiver support programs [33], and/or digital interventions to help manage adherence over the long-term course of rhGH treatment. Although extensive evidence is available in the adult population [34-36], there are few studies addressing the unique needs of digital adherence support in pediatrics [37]. However, the potential of gamified interventions to promote and improve adherence in pediatric patients has recently been demonstrated [38].

### Strengths and Limitations

The strengths of our study include the large data set from a real-world study conducted across 12 countries in one geographic region with substantially diverse and dynamic health care systems, and the fact that the data are derived from a connected injection device, which offers more reliable data compared to data based on the declarations of patients or their parents/caregivers. Comparable insights into patient adherence from other large-scale patient registries for rhGH treatment are not available due to the lack of alternative electronic devices similar to easypod.

Further work is required to assess whether patient/caregiver engagement, as measured by the rate and frequency of data transmission with easypod, is associated with better long-term adherence and clinical outcomes for patients. It would also be interesting to investigate whether or not there is a correlation between the frequency of dose adjustments and the adherence/transmission rate.

Limitations of the study include patient attrition, summarized above, and the fact that the change in adherence rate over time varied from child to child, perhaps due to changes in individual treatment plans or other actions taken by the HCP or child/family. Furthermore, not all data were available for all patients over the same treatment duration, as would be expected in any observational study. Differences between the 12 participating countries in terms of socioeconomic factors, such as reimbursement and/or out-of-pocket expenses for GH therapy or free access to medication, may also have affected individual or local adherence rates. Similarly, differences in prescribing habits (eg, prescription provided for 1, 2, 3, or 6 months), patients’ visits to the clinic where growth response is checked and dose might be adjusted to maximize response to treatment, and refill of prescriptions from country to country may also affect adherence rates. Other potential limitations include the lack of additional information on diagnosis and clinical background, limited data on growth outcomes that do not allow assessment of the full catch-up growth pattern, and lack of patient-reported data such as reasons for discontinuation or interruption of treatment, or predefined actions taken by PSPs [39] in the various countries involved. These data might have been available through linkage to electronic health records (EHRs) where this is permitted, or by allowing patients to have self-reported outcomes entered separately into the system via an app.

Finally, usage of the system (ie, adherence and transmission rates) can be a proxy of usability and utility; however, usage has been to a large extent influenced by the way the system was introduced. All of these areas require more research. Our analysis showed less catch-up growth (–0.17 SD over 24 months) in patients who continue to have low/intermediate adherence compared with patients with high adherence, and this difference increased over time. This difference could be translated into centimeters, resulting in 1.1 cm less catch-up growth between 0-24 months for a patient with an average age for the group (10 years). This is in agreement with the literature, which shows that greater adherence leads to improved growth, with poor adherence adversely affecting growth outcomes [9,10,40]. In the ECOS analyses, statistically significant correlations were observed between adherence and 1-year ∆HSDS (*P*<.001 for patients overall) and height velocity (*P*<.001) [9].

### Future Work

In terms of future research opportunities, analysis of the potential differences between countries with preset additional data collection points would be of interest. The value of this data can be enhanced by complementing it with self-reported height data entered via a patient app. Future research may also allow for automatic self-measurement of height using novel augmented reality technology on mobile phones. Finally, integration with EHRs may facilitate clinical workflows, as has been demonstrated in other therapy areas [16].

### Conclusions

Analysis of the data extracted from easypod Connect showed high adherence to rhGH treatment in Latin American patients, with positive growth outcomes. Thus, our study indicates the potential value of using a connected injection device to monitor and study adherence at an international level. It shows that through our validated method of recording adherence with easypod, we can address an unmet need in rhGH therapy, enabling HCPs to accurately identify patients for whom interventions to improve adherence would be beneficial to improve their growth and other clinical outcomes, particularly as the proportion of children with high adherence declined over time, which is consistent with previous findings. The data also show that children who were most adherent to treatment were more likely to transmit their injection data results regularly and have larger catch-up growth than those who were less adherent. This association between adherence and transmission of data may indicate that sharing data with HCPs has a positive impact on adherence rates, and further studies to confirm this are needed. High adherence and transmission rates may reflect the positive use of the system and could be regarded as indirect indicators of usability and utility of the system by patients and HCPs.

## Abbreviations

ΔHSDS: change in height standard deviation score
ADSS: adherence decision support system
ECOS: Easypod Connect Observational Study
EHR: electronic health record
GH: growth hormone
HCP: health care provider
HSDS: height standard deviation score
PSP: patient support program
rhGH: recombinant human growth hormone

## References

[1] Hommel K, Ramsey R, Rich K, Ryan J, Roberts MC, Steele RG (2017). Adherence to Pediatric Treatment Regimens. Handbook of Pediatric Psychology, Fifth ed.

[2] (2003). Adherence to long-term therapies: evidence for action. World Health Organization.

[3] Cutfield Wayne S, Derraik José G B, Gunn Alistair J, Reid Kyle, Delany Theresa, Robinson Elizabeth, Hofman Paul L (2011). Non-compliance with growth hormone treatment in children is common and impairs linear growth. PLoS One.

[4] Bozzola M, Colle M, Halldin-Stenlid M, Larroque S, Zignani M, easypod™ survey study group (2011). Treatment adherence with the easypod™ growth hormone electronic auto-injector and patient acceptance: survey results from 824 children and their parents. BMC Endocr Disord.

[5] Fisher BG, Acerini CL (2013). Understanding the growth hormone therapy adherence paradigm: a systematic review. Horm Res Paediatr.

[6] Tanaka T, Sato T, Yuasa Mhwm A, Akiyama T, Tawseef A (2021). Patient preferences for growth hormone treatment in Japanese children. Pediatr Int.

[7] Rodríguez Arnao MD, Rodríguez Sánchez A, Díez López I, Ramírez Fernández J, Bermúdez de la Vega JA, Yeste Fernández D, Chueca Guindulain M, Corripio Collado R, Pérez Sánchez J, Fernández González A (2019). Adherence and long-term outcomes of growth hormone therapy with easypod™ in pediatric subjects: Spanish ECOS study. Endocr Connect.

[8] Maggio MC, Vergara B, Porcelli P, Corsello G (2018). Improvement of treatment adherence with growth hormone by easypod™ device: experience of an Italian centre. Ital J Pediatr.

[9] Koledova E, Stoyanov G, Ovbude L, Davies PSW (2018). Adherence and long-term growth outcomes: results from the easypod connect observational study (ECOS) in paediatric patients with growth disorders. Endocr Connect.

[10] van Dommelen P, Koledova E, Wit JM (2018). Effect of adherence to growth hormone treatment on 0-2 year catch-up growth in children with growth hormone deficiency. PLoS One.

[11] Cancela J, Koledova E, Restrepo M (2018). Growth hormone treatment adherence in Latin American patients: Real-world data from the easypod Connect eHealth platform. XXVII Latin American Meeting of Pediatric Endocrinology. Cusco, Peru, October 24-27, 2018: Abstracts. Horm Res Paediatr.

[12] Dunn TC, Xu Y, Hayter G, Ajjan RA (2018). Real-world flash glucose monitoring patterns and associations between self-monitoring frequency and glycaemic measures: A European analysis of over 60 million glucose tests. Diabetes Res Clin Pract.

[13] Calliari LEP, Krakauer M, Vianna AGD, Ram Y, Barbieri DE, Xu Y, Dunn TC (2020). Real-world flash glucose monitoring in Brazil: can sensors make a difference in diabetes management in developing countries?. Diabetol Metab Syndr.

[14] Park S, Sentissi I, Gil SJ, Park W, Oh B, Son AR, Kong YJ, Park S, Paek E, Park YJ, Lee SH (2019). Medication Event Monitoring System for Infectious Tuberculosis Treatment in Morocco: A Retrospective Cohort Study. Int J Environ Res Public Health.

[15] Toscos T, Coupe A, Wagner S, Ahmed R, Roebuck A, Flanagan M, Drouin M, Mirro M (2020). Engaging Patients in Atrial Fibrillation Management via Digital Health Technology: The Impact of Tailored Messaging. J Innov Card Rhythm Manag.

[16] Toscos T, Drouin M, Pater JA, Flanagan M, Wagner S, Coupe A, Ahmed R, Mirro MJ (2020). Medication adherence for atrial fibrillation patients: triangulating measures from a smart pill bottle, e-prescribing software, and patient communication through the electronic health record. JAMIA Open.

[17] Koledova E, Tornincasa V, van Dommelen P (2020). Analysis of real-world data on growth hormone therapy adherence using a connected injection device. BMC Med Inform Decis Mak.

[18] Novillo-Ortiz D, Dumit EM, D'Agostino M, Becerra-Posada F, Kelley ET, Torrent-Sellens J, Jiménez-Zarco A, Saigí-Rubió F (2018). Digital health in the Americas: advances and challenges in connected health. BMJ Innov.

[19] Curioso WH (2019). Building Capacity and Training for Digital Health: Challenges and Opportunities in Latin America. J Med Internet Res.

[20] HealthIT.gov (2018). Clinical Decision Support.

[21] Lion F (2010). Electronic Recording of Growth Hormone Dosing History: The Easypod™ Auto-Injector. CDTH.

[22] de Onis M, Onyango AW, Borghi E, Siyam A, Nishida C, Siekmann J (2007). Development of a WHO growth reference for school-aged children and adolescents. Bull World Health Organ.

[23] WHO Multicentre Growth Reference Study Group (2006). WHO Child Growth Standards based on length/height, weight and age. Acta Paediatr Suppl.

[24] Data Sharing Policy. Merck KGaA.

[25] Dimitri P, Fernandez-Luque L, Banerjee I, Bergadá I, Calliari LE, Dahlgren J, de Arriba A, Lapatto R, Reinehr T, Senniappan S, Thomas-Teinturier C, Tsai M, Anuar Zaini A, Bagha M, Koledova E (2021). An eHealth Framework for Managing Pediatric Growth Disorders and Growth Hormone Therapy. J Med Internet Res.

[26] Lin CY, Updegraff JA, Pakpour AH (2016). The relationship between the theory of planned behavior and medication adherence in patients with epilepsy. Epilepsy Behav.

[27] Cancela J, Guedes S, Koledova E (2018). Real-world data from electronic monitoring of adherence to growth hormone treatment in children with growth disorders: a descriptive analysis.

[28] Bender BG, Ellison MC, Gleason M, Murphy JR, Sundstrom DA, Szefler SJ (2003). Minimizing attrition in a long-term clinical trial of pediatric asthma. Ann Allergy Asthma Immunol.

[29] Cotter R, Burke J, Loeber R, Navratil J (2002). Innovative retention methods in longitudinal research: A case study of the developmental trends study. J Child Fam Stud.

[30] Rodríguez-Arnao M, Sánchez A, López I, Fernández J, Bermúdez de la Vega J, Ballano V, Nieto J, Koledova E (2016). 55th Annual Meeting of the European Society for Paediatric Endocrinology (ESPE), Paris, France, September 10-12, 2016: Abstracts. Horm Res Paediatr.

[31] Ayala-Estrada A, Antillon-Ferreira C, Saavedra-Castillo E, Barrientos-Pérez M, Rivero-Escalante H, Flores-Caloca O, Calzada León R, Valdez-Morales F, Koledova E, Blanco-López A (2016). The Easypod Connect Observational Study (ECOS): Descriptive Analysis of Adherence to Treatment of Growth Hormone Deficient and Small for Gestational Age Naïve to Easypod Patients in Mexico 2012–2015. XXVI Annual Meeting of the Latin American Pediatric Endocrinology Society (SLEP). Buenos Aires, Argentina, November 8-11, 2016: Abstracts. Horm Res Paediatr.

[32] Stoyanov G, Koledova E, Vandermeulen J, the Canadian ECOS group (2016). Objectively measured treatment adherence in the easypod connect observational study (ECOS). Canadian interim analysis: population data and case reports.

[33] Malik S, Moloney C, Koledova E, Reston J, Weinman J (2020). Designing a Personalized Digital Patient Support Program for Patients Treated With Growth Hormone: Key Design Considerations. J Med Internet Res.

[34] Ng R, Carter SR, El-Den S (2020). The impact of mobile applications on medication adherence: a systematic review. Transl Behav Med.

[35] Peng Y, Wang H, Fang Q, Xie L, Shu L, Sun W, Liu Q (2020). Effectiveness of Mobile Applications on Medication Adherence in Adults with Chronic Diseases: A Systematic Review and Meta-Analysis. J Manag Care Spec Pharm.

[36] Pouls BPH, Vriezekolk JE, Bekker CL, Linn AJ, van Onzenoort HAW, Vervloet M, van Dulmen S, van den Bemt BJF (2021). Effect of Interactive eHealth Interventions on Improving Medication Adherence in Adults With Long-Term Medication: Systematic Review. J Med Internet Res.

[37] Ferrante G, Licari A, Marseglia GL, La Grutta S (2021). Digital health interventions in children with asthma. Clin Exp Allergy.

[38] Radovick S, Hershkovitz E, Kalisvaart A, Koning M, Paridaens K, Kamel Boulos MN (2018). Gamification concepts to promote and maintain therapy adherence in children withgrowth hormone deficiency. J Multidiscip Sci.

[39] Seiler P, Guimaraes F, Biglia L, Souza P (2017). PDB14 - Follow-up of pediatric patients under treatment with somatropin in Brazil through a patient support program (PSP). Value in Health.

[40] Takeda A, Cooper K, Bird A, Baxter L, Frampton GK, Gospodarevskaya E, Welch K, Bryant J (2010). Recombinant human growth hormone for the treatment of growth disorders in children: a systematic review and economic evaluation. Health Technol Assess.

